# Structural Analysis and Anti-Complement Activity of Polysaccharides from *Kjellmaniella crsaaifolia*

**DOI:** 10.3390/md13031360

**Published:** 2015-03-16

**Authors:** Wenjing Zhang, Weihua Jin, Delin Sun, Luyu Zhao, Jing Wang, Delin Duan, Quanbin Zhang

**Affiliations:** 1 Institute of Oceanology, Chinese Academy of Sciences, Qingdao 266071, China; E-Mails: wenjingwing@126.com (W.Z.); jinweihua@qdio.ac.cn (W.J.); jingwang@qdio.ac.cn (J.W.); dlduan@qdio.ac.cn (D.D.); 2 College of Earth Science, University of Chinese Academy of Sciences, Beijing 100049, China; 3 Heze Juxinyuan Food Co. Ltd., Heze 274400, China; E-Mails: zhou56018@163.com (D.S.); luyuzhao519@163.com (L.Z.); 4 Nantong Branch, Institute of Oceanology, Chinese Academy of Sciences, Nantong 226006, China

**Keywords:** polysaccharide, *Kjellmaniella crassifolia*, anti-complement

## Abstract

Two polysaccharides, named KCA and KCW, were extracted from *Kjellmaniella crassifolia* using dilute hydrochloric acid and water, respectively. Composition analysis showed that these polysaccharides predominantly consisted of fucose, with galactose, mannose and glucuronic acid as minor components. After degradation and partial desulfation, electrospray ionization mass spectrometry (ESI-MS) was performed, which showed that the polysaccharides consisted of sulfated fucooligosaccharides, sulfated galactofucooligosaccharides and methyl glycosides of mono-sulfated/multi-sulfated fucooligosaccharides. The structures of the oligomeric fragments were further characterized by electrospray ionization collision-induced dissociation tandem mass spectrometry (ESI-CID-MS^2^ and ESI-CID-MS^3^). Moreover, the activity of KCA and KCW against the hemolytic activity of both the classical and alternative complement pathways was determined. The activity of KCA was found to be similar to KCW, suggesting that the method of extraction did not influence the activity. In addition, the degraded polysaccharides (DKCA and DKCW) displayed lower activity levels than the crude polysaccharides (KCA and KCW), indicating that molecular weight had an effect on activity. Moreover, the desulfated fractions (ds-DKCA and ds-DKCW) showed less or no activity, which confirmed that sulfate was important for activity. In conclusion, polysaccharides from *K. crassifolia* may be good candidates for the treatment of diseases involving the complement pathway.

## 1. Introduction

*Kjellmaniella crassifolia* is a brown alga that is widely distributed in the waters around the southern area of Hokkaido, Japan. Compared to other seaweeds, *K. crassifolia* contains high levels of polysaccharides (fucoidan) and the water extract shows very high viscoelasticity. The polysaccharide extract from *K. crassifolia* is an effective immunomodulator and a potent immune adjuvant [1,2]. The structural features of the polysaccharide (fucoidan, not alginate) from *K. crassifolia* have been previously analyzed by extracellular enzyme assays [3] and NMR [4]. The former reported that the novel polysaccharide was fucoglucuronomannan, with a backbone consisting of alternating 4-linked GlcA and 2-linked Man, and was branched at the C-3 position of mannose by a fucopyranose residue. The latter study suggested that the polysaccharide was sulfated fucan, with a backbone of 3-linked fucopyranose sulfated at C-2 and C-4. Because of its accuracy, sensitivity and selectivity, mass spectroscopy (MS) with electrospray ionization (ESI-MS) is an important tool for the analysis of polysaccharides; this technique has been used to successfully elucidate the structures of other heteropolysaccharides [5,6,7].

The complement pathway is an important part of the immune system, playing an essential role in host defense against pathogens. However, inappropriate activation of the complement pathway has been implicated in certain diseases, such as rheumatoid arthritis, Alzheimer’s disease, ischemia-reperfusion injury and systemic lupus erythematosus [8]. Polysaccharides from alga were non-toxic. Numerous natural or semi-synthetic polyanions, such as derivatized dextran, chondroitin sulfate, dextral sulfate, fucoidan and heparin, have been reported to inhibit complement activation [9,10,11,12,13,14].

Recently, *K. crassifolia* was introduced into the Rongcheng coastal area in Shandong Province, China. Thus, in the present study, the structural features of the polysaccharides from *K. crassifolia* cultured in Rongcheng were elucidated by ESI-MS, ESI-CID-MS^2^ and ESI-CID-MS^3^, and the potential activity of the polysaccharides against the classical and alternative complement pathways were investigated.

## 2. Results and Discussion

### 2.1. Preparation of Polysaccharides

The molar ratios of monosaccharides, contents of uronic acid (UA), fucose (Fuc) and sulfate, and average molecular weights of all samples are shown in Table 1. KCA and KCW were found to contain high levels of Fuc together with a small amount of mannose (Man), glucuronic acid (GlcA) and galactose (Gal). With respect to the contents of sulfate and Fuc, KCA contained a higher amount than KCW. In addition, the molecular weight of KCW was higher than KCA, suggesting that KCA degraded slightly during the extraction process. Only trace contents of UA and protein were found. Interestingly, the molecular weight of DKCA was higher than DKCW, indicating that KCW was more sensitive to degradation. Desulfation resulted in a substantial decrease in the molecular weights for both ds-DKCW and ds-DKCA, indicating that the parent compounds were likely to be highly sulfated. In addition, the ratios of other monosaccharides, such as rhamnose (Rha), glucose (Glc) and xylose (Xyl), were increased.

**Table 1:** Chemical composition (%, dry weight) of KCA, KCW and their derived fractions.

Sample	Total Sugar	Fuc (%)	UA (%)	SO_4_ (%)	Protein (%)	Monosaccharides (Molar Ratio)							Mw (kDa)
						Man	Rha	GlcA	Glc	Gal	Xyl	Fuc	
KCW	58.06	37.07	–	24.21	0.45	0.12	0.05	0.09	0.06	0.08	0.02	1	168.7
DKCW	53.56	30.75	–	22.52	0.74	0.12	0.10	0.12	0.06	0.06	–	1	5.2
ds-DKCW	82.42	38.79	3.72	8.93	1.22	0.23	0.11	0.19	0.16	0.21	0.20	1	3.5
KCA	53.40	40.43	–	35.49	–	0.05	0.02	0.06	0.02	0.03	0.02	1	153.7
DKCA	45.76	35.87	–	32.37	–	0.04	0.04	0.05	0.02	0.02	–	1	9.7
ds-DKCA	65.58	48.97	3.51	12.72	0.77	0.13	0.05	0.12	0.27	0.14	0.03	1	2.3

### 2.2. IR Analysis

**Figure 1:**
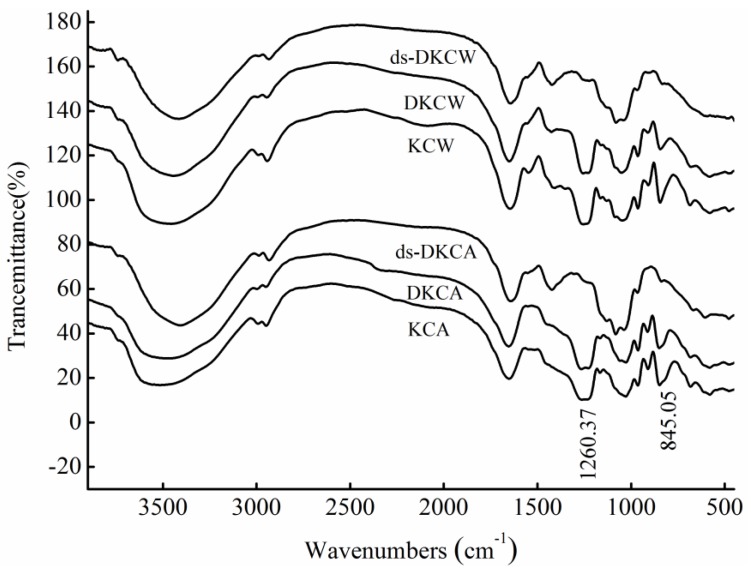
The IR spectra of polysaccharides.

The IR spectra (Figure 1) showed that KCA, DKCA, KCW and DKCW had the same infrared absorption properties, suggesting that they contained the same functional groups. The band at 1260 cm^−1^ corresponded to the S=O stretching vibration, and the band at approximately 845 cm^−1^ was assigned to the C–O–S vibration, suggesting that the presence of the sulfate group was mainly at the C-4 axial position on fucose [15,16,17]. Thus, it was concluded that KCW and KCA were mainly sulfated at C-4 on fucose. After desulfation, the intense band at 1260 cm^−1^ vanished, indicating that ds-DKCW and ds-DKCA lost a high amount of sulfate during the process of desulfation, which was confirmed by the results in Table 1.

### 2.3. MS Analysis of Structure

**Figure 2:**
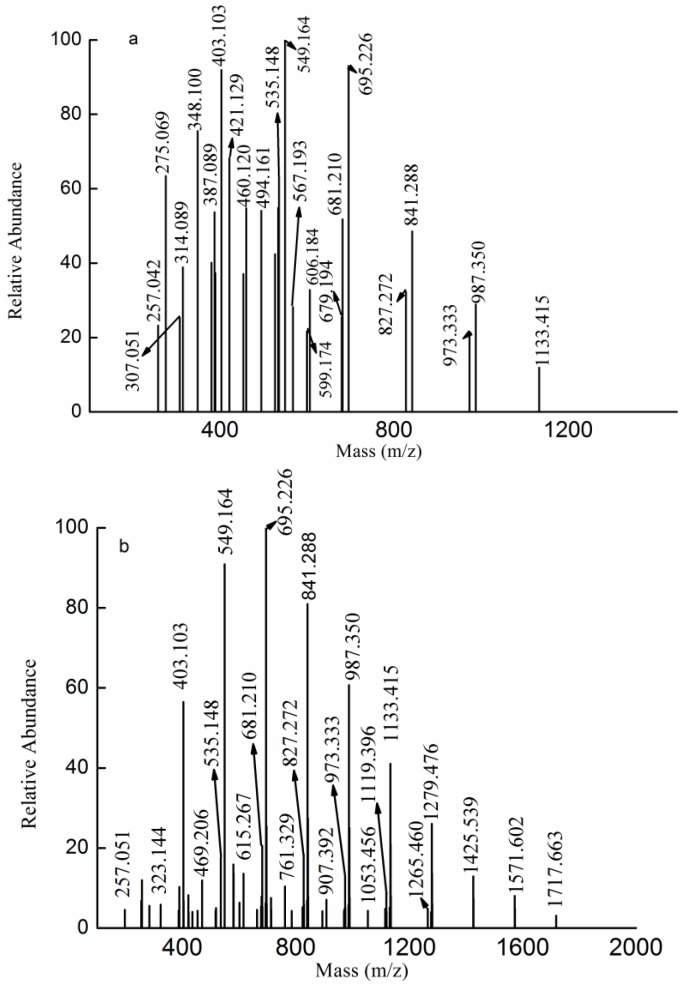
Negative ion mode ESI-MS spectra of ds-DKCW (**a**) and ds-DKCA (**b**).

MS is an important tool for the analysis of heteropolysaccharides because of its speed and sensitivity. Though there have been many studies on the structural features of heteropolysaccharides [5,6,18,19,20,21,22,23], it was previously not possible to analyze heteropolysaccharides that contained large, highly charged molecules. Thus, heteropolysaccharides needed to be degraded. The ESI-MS spectrum of ds-DKCW determined in the present study was shown in Figure 2a. There were five types of fragment ions. The most intense peaks at *m/z* 257.042, 403.103, 549.164, 695.226, 841.288, 987.350 and 1133.415 were determined to be [MeFuc_n_SO_3_Na-Na]^−^ (*n* = 1–7). The less intense fragment ions at *m/z* 389.089, 535.148, 681.210, 827.272 and 973.333 revealed a distribution of singly charged ions, corresponding to sulfated fucooligosaccharides [Fuc_n_SO_3_Na-Na]^−^ (*n* = 2–6). In addition, the set of fragment ions at *m/z* 314.089, 387.089, 460.120, 533.148, 606.184 and 679.194 was determined to be methyl glycosides of di-sulfated fucooligosaccharides [MeFuc_n_(SO_3_Na)_2_-2Na]^2−^ (*n* = 3–8), and the fragment ions at *m/z* 307.051, 380.089, 453.112, 526.141 and 599.174 corresponded to di-sulfated fucooligosaccharides [Fuc_n_(SO_3_Na)_2_-2Na]^2−^ (*n* = 3–7). Finally, the last set of doubly charged ions at *m/z* 275.069, 348.100, 421.129, 494.161 and 567.193 was determined to be [Gal(Fuc)_n_SO_3_Na-Na]^2−^ (*n* = 2–7) (the hexose was determined to be D-Gal based on the analysis of monosaccharides).

The ESI-MS spectrum of ds-DKCA is shown in Figure 2b. The fragment ions were similar to those of ds-DKCW. However, ds-DKCA mainly consisted of methyl glycosides of mono-sulfated fucooligosaccharides and had few multi-sulfated fucooligosaccharides or sulfated galactofucooligosaccharides.

**Figure 3:**
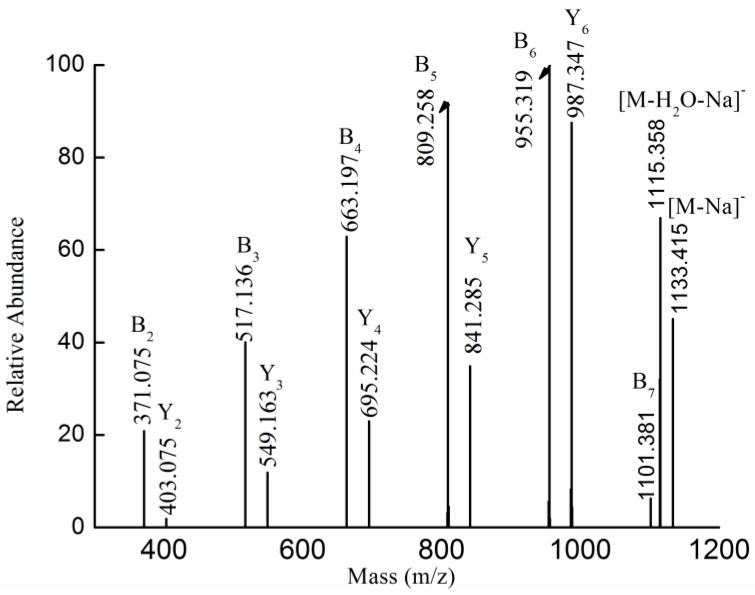
Negative ion mode ESI-CID-MS^2^ spectrum of the ion [MeFuc_7_SO_3_Na-Na]^−^ at *m/z* 1133.415 (−1).

The fragmentation pattern for the singly-charged ion at *m/z* 1133.415 (−1), assigned as [MeFuc_7_SO_3_Na-Na]^−^, was displayed in Figure 3. Singly-charged fragment ions at *m/z* 371.075, 517.136, 663.197, 809.258, 955.319 and 1101.381 corresponded to B-type ions, arising from the glycosidic bond cleavage from the reducing end, suggesting that the sulfate was located at the non-reducing end. Another set of fragment ions at *m/z* 403.075, 549.063, 841.285 and 987.347 (a loss of fucopyranose residue (146 Da)) arose from the glycosidic bond cleavage from the non-reducing end, indicating that the sulfate was located at the reducing end. Thus it was concluded that the ion [MeFuc_7_SO_3_Na-Na]^−^ at *m/z* 1133.415 (−1) was a mixture of isomers Fuc(SO_3_Na)→Fuc→Fuc→Fuc→Fuc→Fuc→Fuc-OMe and Fuc→Fuc→Fuc→Fuc→Fuc→Fuc→Fuc(SO_3_Na)-OMe. This finding was in agreement with previous studies on the structural features of polysaccharides from *Saccharina japonica* and *Sargassum fusiforme* [22,23,24], suggesting that polysaccharides from *K. crassifolia* might have the same backbone as the fucoidan from *Saccharina japonica* and *Sargassum fusiforme*.

**Figure 4:**
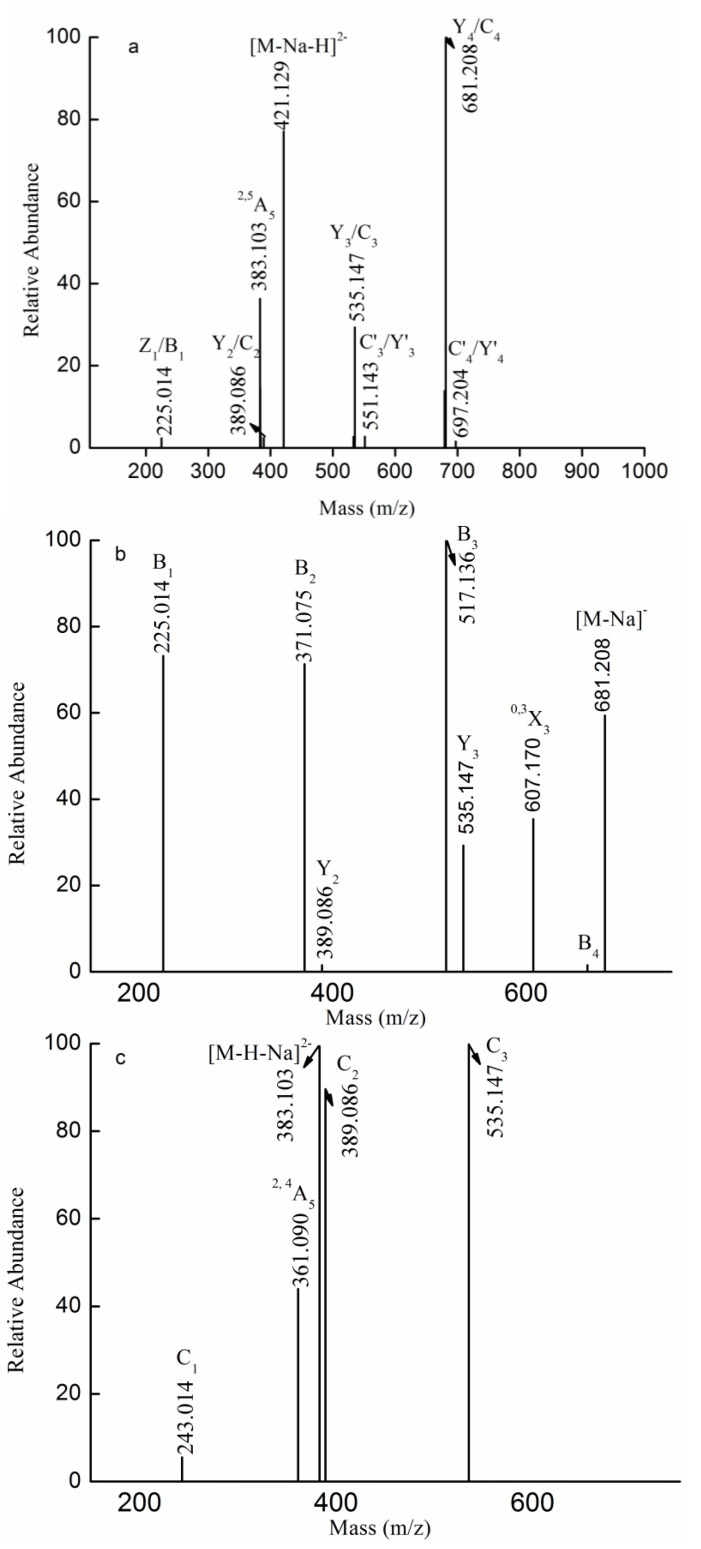
Negative ion mode ESI-CID-MS^2^ spectrum of the ion at *m/z* 421.129 (−2) (**a**) and negative ion mode ESI-CID-MS^3^ spectra of the ions at *m/z* 681.208(−1) (**b**) and 383.103 (−2) (**c**).

The fragmentation pattern for the doubly charged ion at *m/z* 421.129 (−2) assigned to the ion [Gal(Fuc)_4_SO_3_Na-Na-H]^2−^ was displayed in Figure 4a. The fragment ion at *m/z* 225.014 resulted from breakage of glycosidic bonds and cleavage of the dehydrated, sulfated fucose residue. No ions at *m/z* 241 or 259 were detected, suggesting that the sulfate group was substituted at the Fuc residue. One set of fragment ions at *m/z* 389.086, 535.147 and 681.208, assigned as C- or Y-type fragment ions, corresponded to [(Fuc)_2_SO_3_Na-Na]^−^, [(Fuc)_3_SO_3_Na-Na]^−^ and [(Fuc)_4_SO_3_Na-Na]^−^, respectively. Another series of less intense fragment ions at *m/z* 551.143(C'_3_/Y'_3_) and 697.204(C'_4_/Y_4_) corresponded to [Gal(Fuc)_2_SO_3_Na-Na]^−^ and [Gal(Fuc)_3_SO_3_Na-Na]^−^, respectively. In addition, the characteristic fragment ion at *m/z* 383.103 (−2) corresponded to ^2,5^A_5_, suggesting that the linkage between Fuc and Gal was a 1→4 linkage or a 1→3 linkage. Moreover, no characteristic ion of ^0,2^A was detected, suggesting that the linkage between Fuc and Fuc was mainly 3-linked. Thus, it was hypothesized that Gal(Fuc)_4_SO_3_Na consisted of Fuc(SO_3_Na)→Fuc→Fuc→Fuc→Gal, Fuc(SO_3_Na)→Fuc→Fuc→Gal→Fuc, Fuc(SO_3_Na)→Fuc→Gal→Fuc→Fuc, Fuc(SO_3_Na)→Gal→Fuc→Fuc→Fuc, Fuc→Gal→Fuc→Fuc→ Fuc(SO_3_Na), Gal→Fuc→Fuc→Fuc→Fuc(SO_3_Na), Fuc→Fuc→Gal→Fuc→Fuc(SO_3_Na) and Fuc→Fuc→Fuc→Gal→Fuc(SO_3_Na).

To confirm the above hypothesis, the fragmentation pattern for the singly-charged ion at *m/z* 681.208 was characterized by ESI-CID-MS^3^ and was found to correspond to the ion [Fuc_4_SO_3_Na-Na]^−^, as shown in Figure 4b. One set of fragment ions at 225.014, 371.075 and 517.136 assigned as B-type ions arose from the glycosidic bond cleavage from the reducing end, suggesting that the sulfate was located at the non-reducing end. Anastyuk *et al*. and Saad and Leary [18,25] reported that, when an oligosaccharide’s sulfate group was close to the glycosidic linkage spatially, it would undergo easier B-type fragmentation, which indicated that the sulfate group was substituted at C-2. Another set of less intense fragment ions, 389.086 and 535.147, corresponded to Y-type ions, suggesting the sulfate was substituted at the reducing end. In addition, the characteristic ion at *m/z* 607.170 was a ^0,3^X_3_-type ion, suggesting that the linkage was a 1→3 linkage. No ^0,2^A-type ions were detected, which confirmed that the linkage between Fuc and Fuc was 3-linked. In sum, Fuc_4_SO_3_Na consisted primarily of Fuc(2SO_3_Na)→Fuc→Fuc→Fuc, and, to a lesser degree, of Fuc→Fuc→Fuc→Fuc(2 or 4SO_3_Na).

The fragmentation pattern for the doubly charged ion at *m/z* 383.103 (−2) was also elucidated by ESI-CID-MS^3^ in Figure 4c. The characteristic ion at *m/z* 361.090 (−2) (^2,4^A_5_) arose from the loss of C_2_H_4_O (44 Da) from the ion at *m/z* 383.103 (−2) (^2,5^A_5_), suggesting that the reducing end was a Gal residue. In addition, it also indicated that the linkage between Fuc and Gal was a 1→4 linkage. Moreover, a set of fragment ions at *m/z* 243.014, 389.086 and 535.147 were determined to be C-type ions, suggesting that the sulfate was located at the non-reducing end. This glycosidic cleavage observation was not consistent with the previous study [18,25], indicating that the sulfate was at C-4. Therefore, it was concluded that Gal(Fuc)_4_SO_3_Na was Fuc(4SO_3_Na)→Fuc→Fuc→Fuc→Gal.

Thus, it was concluded that Gal(Fuc)_4_SO_3_Na was mainly made of Fuc(2 or 4SO_3_Na)→Fuc→Fuc→Fuc→Gal. In addition, it also might contain Gal→Fuc→Fuc→Fuc→Fuc(2 or 4SO_3_Na), Fuc(SO_3_Na)→Fuc→Fuc→Gal→Fuc, Fuc(SO_3_Na)→Fuc→Gal→Fuc→Fuc, Fuc(SO_3_Na)→Gal→Fuc→Fuc→Fuc, Fuc→Gal→Fuc→Fuc→Fuc(SO_3_Na), Fuc→Fuc→Gal→Fuc→Fuc(SO_3_Na) and Fuc→Fuc→Fuc→Gal→Fuc(SO_3_Na).

**Figure 5:**
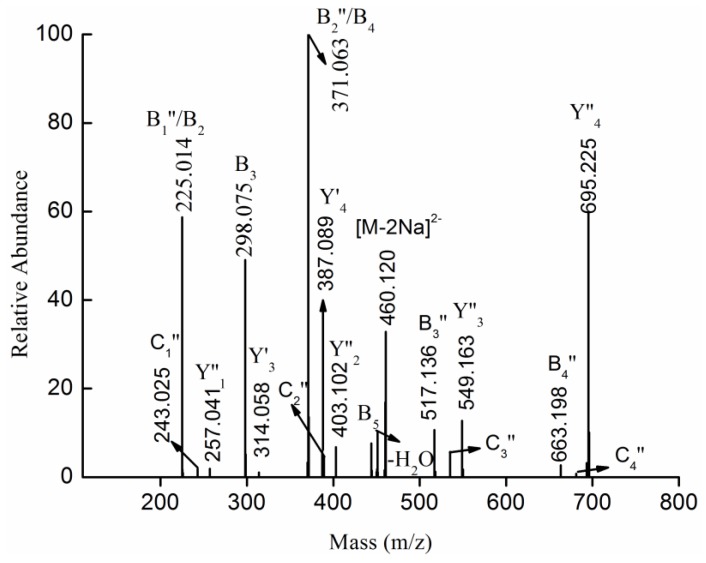
Negative ion mode ESI-CID-MS^2^ spectrum of the ion [MeFuc_5_(SO_3_Na)_2_-2Na]^2−^ at *m/z* 460.120 (−2).

The fragmentation for the doubly charged ion at *m/z* 460.120 (−2), corresponding to the ion [MeFuc_5_(SO_3_Na)_2_-2Na]^2−^, was shown in Figure 5. Five types of fragments ions were found: (1) doubly charged fragment ions at *m/z* 225.014 (B_2_), 298.075 (B_3_), 371.063 (B_4_) and 444.106 (B_5_) corresponded to [Fuc_2_(SO_3_Na)_2_-2Na]^2−^, [Fuc_3_(SO_3_Na)_2_-2Na]^2−^, [Fuc_4_(SO_3_Na)_2_-2Na]^2−^ and [Fuc_5_(SO_3_Na)_2_-2Na]^2−^, respectively. No fragment ion at *m/z* 152 ([Fuc(SO_3_Na)_2_-2Na]^2−^) was observed, suggesting that one isomer of MeFuc_5_(SO_3_Na)_2_ was Fuc(SO_3_Na)→Fuc(SO_3_Na)→Fuc→Fuc→Fuc-OMe; (2) Less intense, doubly charged fragment ions at *m/z* 314.058 (Y_3_') and 387.089 (Y_4_') were determined to be [MeFuc_3_(SO_3_Na)_2_-2Na]^2−^ and [MeFuc_4_(SO_3_Na)_2_-2Na]^2−^, most likely having arisen from glycosidic bond cleavage at the non-reducing terminus. Thus, it was hypothesized that MeFuc_5_(SO_3_Na)_2_ contained Fuc→Fuc→Fuc→Fuc(SO_3_Na)→Fuc(SO_3_Na)-OMe; (3) Singly charged fragment ions at *m/z* 225.014, 371.063, 517.136 and 663.198 (namely, B_1_'', B_2_'', B_3_'' and B_4_'', respectively) arose from the loss of a methyl glycoside of sulfated fucose (257 Da) from the reducing terminus, suggesting that MeFuc_5_(SO_3_Na)_2_ might consist of Fuc(SO_3_Na)→Fuc→Fuc→Fuc→Fuc(SO_3_Na)-OMe or Fuc→Fuc→Fuc→Fuc(SO_3_Na)→Fuc(SO_3_Na)-OMe; (4) A singly-charged fragment ion at *m/z* 695.225 (namely, Y_4_''), assigned as [MeFuc_4_SO_3_Na-Na]^−^, arose from the loss of a sulfated fucopyranose residue (225 Da) from the side chain or from the non-reducing terminus. A singly-charged fragment ion at 549.163 (namely, Y_3_''), corresponding to [MeFuc_3_SO_3_Na-Na]^−^, arose from the loss of fucopyranose after the loss of the sulfated fucopyranose. Thus it was hypothesized that MeFuc_5_(SO_3_Na)_2_ was made up of Fuc(SO_3_Na)→Fuc→Fuc→Fuc→Fuc(SO_3_Na)-OMe, Fuc(SO_3_Na)→Fuc(SO_3_Na)→ Fuc→Fuc→Fuc-OMe and Fuc→Fuc→Fuc→Fuc(SO_3_Na)→Fuc(SO_3_Na)-OMe; (5) Singly-charged fragment ions at *m/z* 243.025 and 389.086 (namely, C1''and C2'') arose from the loss of sulfated fucose from the non-reducing terminus, suggesting that MeFuc_5_(SO_3_Na)_2_ might consist of Fuc(SO_3_Na)→Fuc→Fuc→Fuc→Fuc(SO_3_Na)-OMe. Thus, it was hypothesized that MeFuc_5_(SO_3_Na)_2_ was made up of Fuc(SO_3_Na)→Fuc→Fuc→Fuc→Fuc(SO_3_Na)-OMe, Fuc(SO_3_Na)→Fuc(SO_3_Na)→Fuc→Fuc→Fuc-OMe and Fuc→Fuc→Fuc→Fuc(SO_3_Na)→Fuc(SO_3_Na)-OMe.

**Figure 6:**
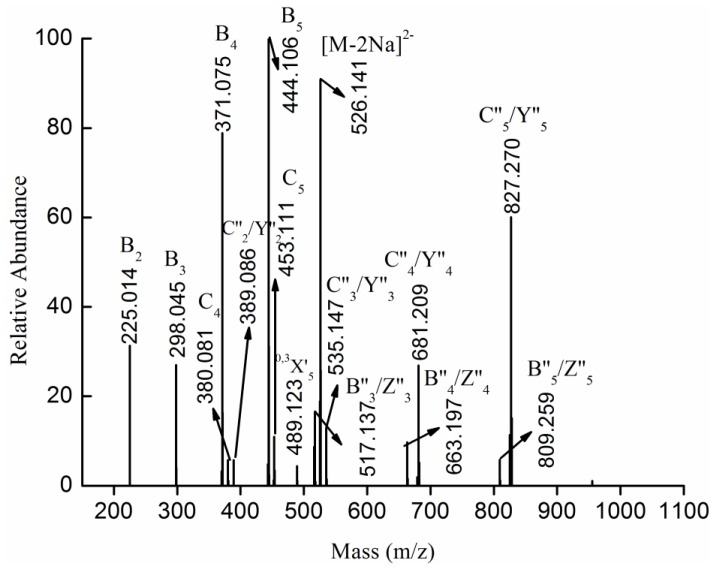
Negative ion mode ESI-MS^2^ spectrum of ion [Fuc_6_(SO_3_Na)_2_-2Na]^2−^ at *m/z* 526.141 (−2).

The fragmentation pattern for the doubly charged ion at *m/z* 526.141 (−2), assigned to the ion [Fuc_6_(SO_3_Na)_2_-2Na]^2−^, was displayed in Figure 6. The ion at *m/z* 526.141 (−2) was the prototype of the ion [MeFuc_6_(SO_3_Na)_2_-2Na]^2−^, which was confirmed by its similar fragmentation pattern. In other words, both the ion [MeFuc_6_(SO_3_Na)_2_-2Na]^2−^ and the ion [Fuc_6_(SO_3_Na)_2_-2Na]^2−^ were generated under the condition of desulfation. However, the former was methylated, while the latter was not.

### 2.4. Anti-Complement Activity

**Figure 7:**
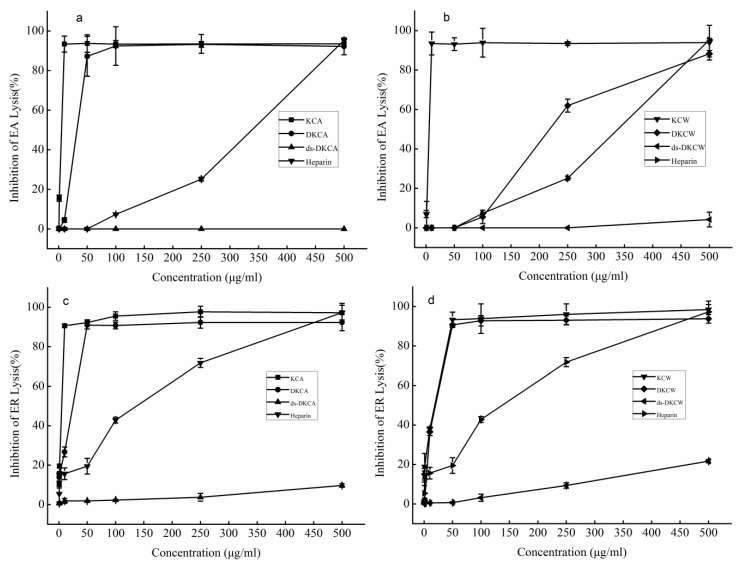
Inhibition of the classical pathway-mediated hemolysis of EA (**a** and **b**) and alternative pathway-mediated hemolysis of ER (**c** and **d**) in 1:10-diluted NHS in the presence of increasing amounts of the polysaccharides. Heparin was used as the reference. The results are expressed as percent inhibition of hemolysis. Data are the means from 3 determinations ± S.E.M.

As shown in Figure 7a–d, the effects of the polysaccharides on activation of human complement through the classical pathway (Figure 7a,b) and the alternative pathway (Figure 7c,d) were examined in 1:10-diluted NHS, with heparin used as a reference. The complement group (*i.e*., positive control) displayed a 93.11% ± 2.96% activation of the classical complement pathway. The activities of KCA, KCW, DKCA, DKCW and heparin were dose-dependent, while ds-DKCA and ds-DKCW showed little or no activity (Figure 7a,b). The activities of KCA and KCW reached a plateau at a concentration of 10 μg/mL, while DKCA plateaued at 50 μg/mL. In addition, the concentration that resulted in 50% inhibition of the classical complement pathway (CH_50_) for DKCW was approximately 218 μg/mL, which was lower than heparin. Therefore, KCA, KCW, and DKCA were more potent than heparin in inhibiting activation of the classical pathway. On the other hand, the concentrations of KCA, KCW, DKCA, DKCW and heparin that resulted in 50% inhibition of the alternative pathway (AP50) were 4.83, 18.60, 24.50, 19.97 and 137.25 μg/mL, respectively (Figure 7c,d). This finding indicated that KCA, KCW, DKCA and DKCW were more potent than heparin in inhibiting activation of the alternative pathway.

KCA and KCW displayed similar activity levels against the two complement pathways, indicating that the extraction methods did not affect the activity levels. The degraded polysaccharides DKCA and DKCW exhibited weaker activity compared to the crude polysaccharides KCA and KCW, which suggested, as others have reported, that the change in molecular weight influenced the anti-complement activity of the two compounds [9,26]. In addition, the finding that ds-DKCA and ds-DKCW showed little or no activity against the two pathways suggested that sulfate was important for anti-complement activity, which was in agreement with other reports [13,27,28].

In sum, the polysaccharides from *Kjellmaniella crassifolia* may be potent drugs that are capable of suppressing complement activation.

## 3. Experimental Section

### 3.1. Preparation of Polysaccharides

*K. crassifolia* was collected in Rongcheng, Shandong Province, China, in June of 2013. The polysaccharides were extracted from *K. crassifolia* as previously described [29]. Briefly, the dried algae were cut into pieces, and the polysaccharides were extracted three times in water at room temperature for 2 h. The solution was dialyzed against water and distilled water. Finally, the polysaccharide was concentrated and precipitated with ethanol. The resultant precipitate was named KCW. In an alternative extraction, the dried algae were cut and extracted with 0.1 M HCl at room temperature for 2 h. The solution was neutralized, concentrated, dialyzed and precipitated with ethanol. This precipitate was named KCA.

### 3.2. Preparation of Low Molecular Weight Fucoidans and Their Desulfated Mixtures

The crude polysaccharides KCA and KCW were degraded using hydrogen dioxide and ascorbic acid to obtain low molecular weight polysaccharides, as previously described [21]. Briefly, crude polysaccharide (1 g) was dissolved in water (100 mL). Ascorbic acid (0.5 g) and hydrogen dioxide (0.3 mL) were then added, and the solution was stirred for 2 h at room temperature. The degraded polysaccharides (e.g., DKCA and DKCW) were obtained after ultrafiltration, concentration and lyophilization.

The desulfation of DKCA and DKCW was performed according to the modified method of Nagasawa *et al.* [30]. Desulfation was carried out using its pyridinium salt. Briefly, the sample was dissolved in distilled water (10 mL) and mixed with cationic resin for 3 h. After filtration, the solution was neutralized with pyridinium and lyophilized. The polysaccharide was then dissolved in 20 mL of a 9:1 ratio of dimethyl sulfoxide: methanol (*v*:*v*) at 80 °C for 5 h. The desulfated solution was dialyzed and lyophilized to give desulfated products (e.g., ds-DKCA and ds-DKCW).

### 3.3. Composition Methods

Total sugar and fucose content were determined according to the method of Dubois *et al*. [31] and Gibbons [32], using fucose as a standard. The level of sulfation was analyzed by the barium chloride-gelatin method of Kawai *et al*. [33]. The uronic acid (UA) content was estimated with a modified carbazole method using d-glucuronic acid as a standard [34]. The protein level was determined according to the method of Bradford *et al*. [35]. For the determination of sugar composition, the acid-hydrolyzed glycoses were converted into their 1-phenyl-3-methyl-5-pyrazolone derivatives (PMP) and separated by HPLC chromatography [36]. The molecular weight of the samples was assayed by a HP-GPC system on a TSK gel PWxl 3000 column (7 μm, 7.8 × 300 mm) eluted with 0.2 M Na_2_SO_4_ at a flow rate of 0.5 mL min^−1^ at 30 °C [37].

### 3.4. Spectroscopic Analysis

Infrared spectra (IR) were recorded from polysaccharide powder in KBr pellets on a Nicolet-360 FTIR spectrometer between 400 and 4000 cm^−1^ (36 scans, at a resolution of 6 cm^−1^).

MS was performed on a LTQ ORBITRAR XL (Thermo Scientific, Waltham, MA, USA). Samples, dissolved in CH_3_CN-H_2_O (1:1, *v*:*v*), were introduced into the MS at a flow rate of 5 μL min^−1^ in the negative ionization mode. The capillary voltage was set to −3000 V, the cone voltage was set to −50 V, the source temperature was set to 80 °C, and the desolvation temperature was set to 150 °C. The collision energy was optimized between 20 and 50 eV. All spectra were analyzed by Xcalibur (Thermo Scientific, Waltham, MA, USA).

### 3.5. Anti-Complement Activity

The anti-complement activity of the polysaccharides was determined by measuring their ability to inhibit classical [38] and alternative [39] complement-mediated hemolysis. For the classical pathway, 100 μL of various dilutions of tested samples were mixed with 100 μL of 1:10-diluted normal human serum (NHS, which was obtained from healthy, adult donors), 200 μL GVB^2+^ (veronal buffer saline (VBS) containing 0.1% gelatin, 0.5 mM Mg^2+^ and 0.15 mM Ca^2+^) and 200 μL of sensitized erythrocytes (EA). The mixture was then incubated at 37 °C for 30 min. The following assay controls were incubated under the same conditions: (1) 100% lysis: 200 μL of EA in 400 of μL water; (2) sample control: 100 μL of sample in 500 of μL GVB^2+^; (3) complement: 100μL of 1:10-diluted NHS and 200 μL EA in 300 μL GVB^2+^; and (4) blank: 200 μL of EA in 400 μL of GVB^2+^. After incubation, the mixture was centrifuged (5000 rpm × 10 min) and the erythrocyte lysis was determined at 405 nm. Decreased lysis in the presence of tested polysaccharides indicated anti-complement activity. All of the samples were dissolved in GVB^2+^. Heparin sodium salt (Beijing Rui Taibio Co., Ltd., Beijing, China), at a concentration of 160 IU/mg, was used as the reference (The molecular weight was 54.1 kDa and sulfate content was 13.60%). The inhibition percentage was calculated using the following equation: inhibition of EA lysis (%) = (A_complement_ − [A_sample_ − A_sample control_])/A_complement_ × 100.

To assay sample inhibition of the alternative complement pathway, 150 μL of various dilutions of tested samples were mixed with 150 μL of 1:10-diluted NHS and 200 μL of rabbit erythrocytes (ER). The mixture was then incubated for 30 min at 37 °C. Cell lysis was determined by the same method as described above for the classical pathway. Controls for 100% lysis, sample control, complement and blank were included. The percent inhibition was calculated using the following equation: inhibition of ER lysis (%) = (A_complement_ − [A_sample_ − A_sample control_])/A_complement_ × 100.

## 4. Conclusions

In this study, two polysaccharides, KCW and KCA, were extracted from *K. crassifolia* using water and dilute hydrochloric acid, respectively, and their degraded fractions and desulfated fractions were prepared. The chemical compositions indicated that KCA had higher contents of sulfate and Fuc and less of other monosaccharides than KCW. To elucidate the structural features of KCA and KCW, they were degraded and partly desulfated. The desulfated mixtures were determined by ESI-MS. Both were found to contain sulfated fucooligosaccharides, sulfated galactofucooligosaccharides and methyl glycosides of mono-sulfated/multi-sulfated fucooligosaccharides. The major difference between ds-DKCA and ds-DKCW was the intensity of the fragment ions. In addition, the structural features of oligomeric fragments were characterized by ESI-CID-MS^2^ and ESI-CID-MS^3^. It was shown that the polysaccharides had a backbone of 3-linked Fuc residues sulfated at C-2, C-4 or C-2 and C-4. Some oligomers had the 4-linked Gal residue at the reducing terminus. Some fucooligomers were interspersed by Gal residues. Moreover, activities of the polysaccharides against the classical and alternative complement pathways were measured. The crude polysaccharides KCA and KCW had the highest activity levels, while the desulfated fractions ds-DKCA and ds-DKCW had little or no activity. These data suggested that the change in molecular weight and sulfate content influenced the activity levels. In summary, polysaccharides from *K. crassifolia* may be a good candidate drug for anti-complement therapy.
